# Supplementary figures and images for: Identification of a New HIV-1 BC Intersubtype Circulating Recombinant Form (CRF108_BC) in Spain
Source: Viruses. 2021 Jan 12;13(1):93. doi: 10.3390/v13010093 (PMC7826730; doi:10.3390/v13010093)

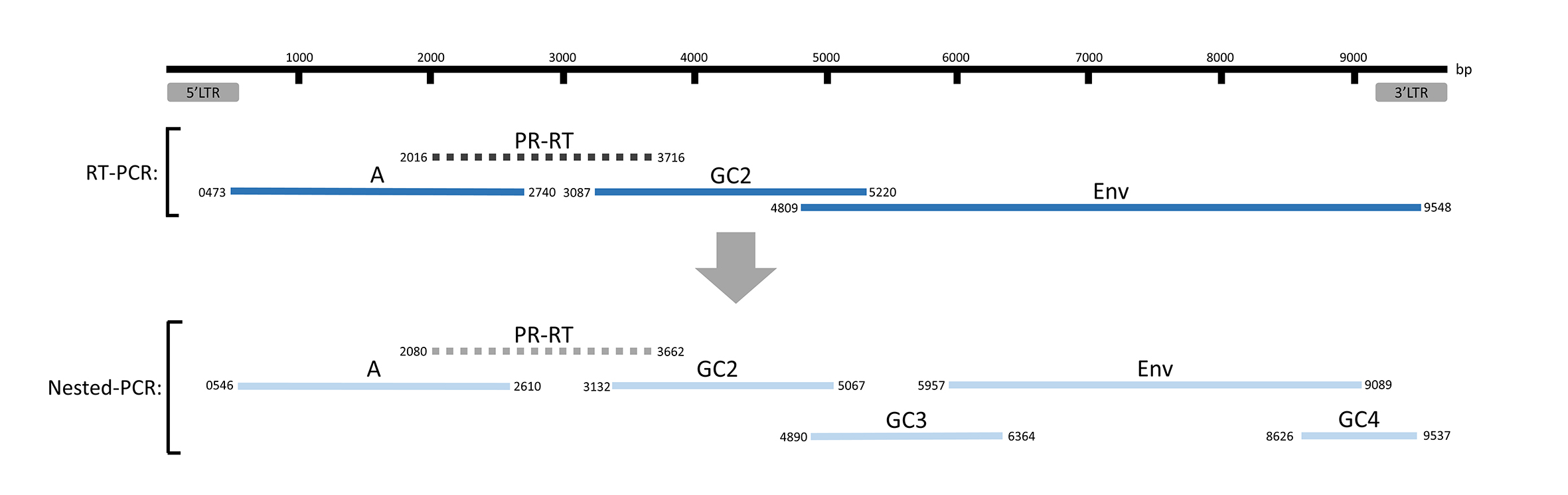

Supplement: Supplementary file 1 [file viruses-13-00093-s001.zip › supplementary/Supplementary_Fig1_300dpi_20cm.jpg]

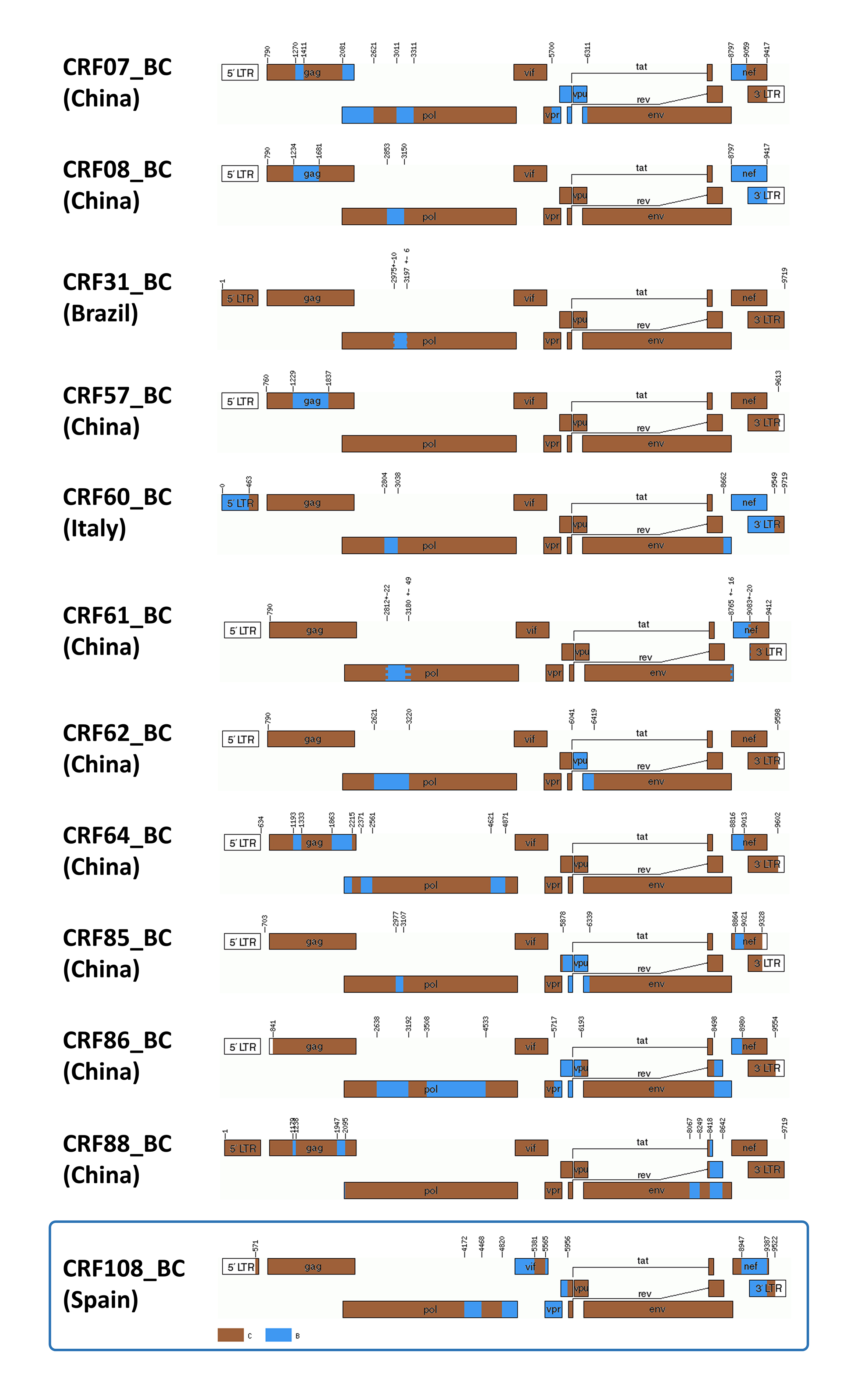

Supplement: Supplementary file 1 [file viruses-13-00093-s001.zip › supplementary/Supplementary_Fig2_300dpi_20cm.jpg]

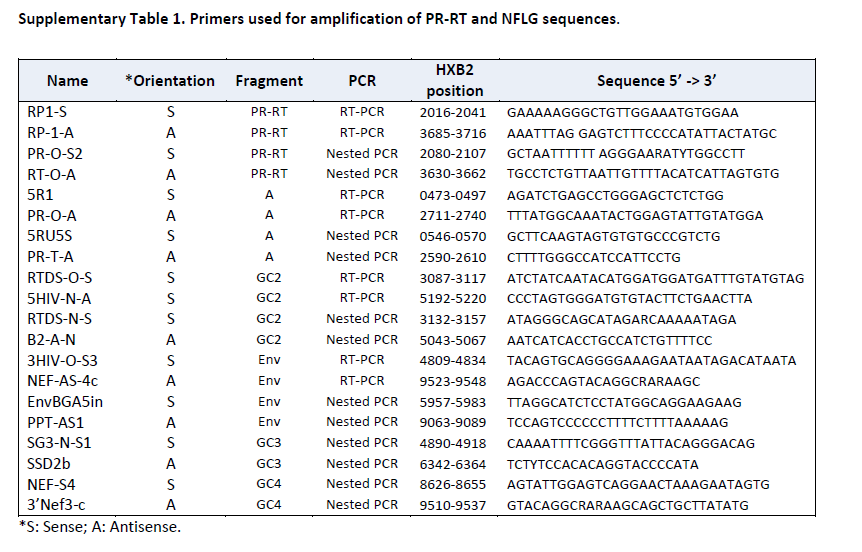

Supplement: Supplementary file 1 [file viruses-13-00093-s001.zip › supplementary/table s1.png]
